# Orchestrating innate immunity through RNA editing and helicase activity: ADAR1, dsRNA sensors, and tumor immune evasion

**DOI:** 10.3389/fcell.2026.1788799

**Published:** 2026-04-14

**Authors:** Moeko Minakuchi, Joseph M. Salvino, Jessie Villanueva

**Affiliations:** Molecular and Cellular Oncogenesis Program, Ellen and Ronald Caplan Cancer Center, The Wistar Institute, Philadelphia, PA, United States

**Keywords:** ADAR1, dsRNA sensors, helicase activity, RNA editing, tumor immune evasion

## Abstract

Dysregulated dsRNA editing and RNA metabolism in cancer contribute to immune evasion, highlighting the critical role of RNA structural regulation in disease. Intracellular RNA structures regulate gene expression, innate immunity, genome stability, and cell fate. Among these, double-stranded RNA (dsRNA) is particularly important; exogenous dsRNA typically originates from viral infection, whereas endogenous dsRNA arises from repetitive elements or transcriptional errors, allowing cells to distinguish “self” from “non-self.” The RNA-editing enzyme Adenosine Deaminase Acting on RNA 1 (ADAR1) prevents inappropriate innate immune activation by catalyzing adenosine-to-inosine (A-to-I) editing of endogenous dsRNA. RNA helicases complement this function by remodeling RNA structures and resolving nucleic acid hybrids, maintaining RNA homeostasis and immune surveillance. Recent studies have revealed an interplay between ADAR1 and RNA helicases that regulate dsRNA immunogenicity and R-loop dynamics, establishing this network as a key determinant of tumor immunity. Dysregulated RNA editing and structural regulation in cancer further underscore the potential of targeting these pathways therapeutically, providing strategies beyond conventional gene- or protein-centered approaches. In this review, we summarize current insights into how ADAR1 and RNA helicases control RNA structure, emphasize their roles in innate immune sensing, and discuss emerging approaches to modulate RNA editing and RNA architecture for therapeutic benefit. Taken together, research in this area positions RNA structural control as a central determinant of immune homeostasis and a promising frontier in cancer therapy.

## 1 Introduction

**FIGURE 1:**
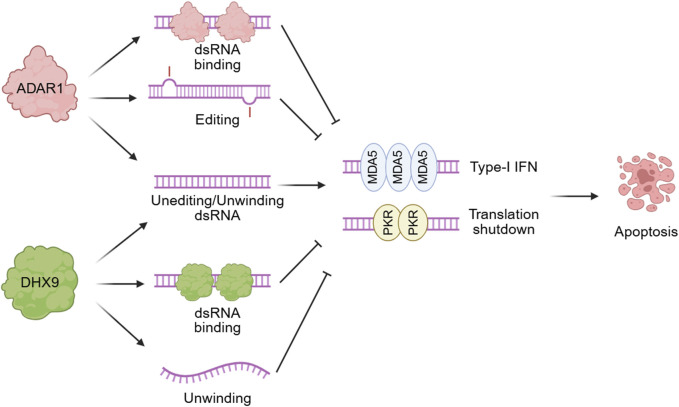
The dsRNA-mediated innate immune response regulated by ADAR1 or DHX9. Endogenous dsRNA localized in the cytoplasm binds to ADAR1p150 and/or DHX9 and undergoes editing, thereby evading recognition by the dsRNA sensors and innate immune receptors PKR and MDA5.

RNA is more than just genetic information between DNA and proteins. Its structural versatility enables a wide range of functions within the cell. Although inherently single-stranded, RNA molecules can fold into intricate secondary structures through intra-molecular base pairing (Wan et al., 2011). These conformations not only influence gene expression, translational efficiency, RNA stability, and subcellular localization, but also play integral roles in maintaining cellular homeostasis, orchestrating stress responses, and guiding cell fate decisions (Wan et al., 2011; Dethoff et al., 2012). Among these structural elements, dsRNA has emerged as a critical signaling molecule. While traditionally associated with exogenous viral RNAs, recent studies have revealed that dsRNA can also arise endogenously from repetitive elements, transcriptional dysregulation, and the accumulation of reverse transcribed products (Mu et al., 2016; Chen and Hur, 2022; Kassiotis, 2023). Endogenous dsRNA is recognized as “self” to prevent excessive innate immune activation (Chen and Hur, 2022). This recognition is mainly mediated through ADAR1-mediated A-to-I editing and regulation of dsRNA sensing pathways (Pestal et al., 2015; Schlee and Hartmann, 2016; Rice et al., 2012; Liddicoat et al., 2015). The secondary structures of dsRNAs influence interactions with ADAR1 and sensing molecules, thereby contributing to self/non-self-discrimination. The dynamic remodeling of RNA structures via A-to-I editing by ADAR1 or unwinding by RNA helicases enables the modulation of immunogenicity and the fine-tuning of gene regulatory networks (Liddicoat et al., 2015; Nishikura, 2016; Linder and Jankowsky, 2011; Mannion et al., 2014; Rehwinkel and Gack, 2020). Although the precise identity of immunogenic dsRNAs has not yet been fully established, recent studies suggest that a restricted subset of dsRNAs, for which ADAR1-mediated editing is particularly important, contributes to Melanoma differentiation-associated protein 5 (MDA5)-dependent responses (Sun T. et al., 2025). Given that MDA5 preferentially binds long, continuous dsRNA structures, whereas Protein kinase R (PKR) is less dependent on dsRNA length and can be activated by localized double-stranded regions (Chen and Hur, 2022), the RNA species responsible for activating each sensor may not be identical (Cottrell et al., 2024a). Here, “immunogenic dsRNA” refers to cytosolic dsRNA species that can activate innate immune sensors such as MDA5 (encoded by *IFIH1*) and PKR (encoded by *EIF2AK2*). This review focuses on two principal regulators of RNA structural dynamics: ADAR1-mediated RNA editing and RNA helicase-mediated unwinding, emphasizing their roles in shaping the intracellular RNA landscape and coordinating immune surveillance (Figure 1).

## 2 Overview of ADAR1-mediated RNA editing

ADAR1 catalyzes the conversion of adenosine (A) to inosine (I) within dsRNA, with inosine read as guanosine (G) by ribosomes and reverse transcriptase. While ADAR1 can edit coding regions, ADAR2 primarily mediates site-specific recoding, whereas ADAR1 mainly targets non-coding repetitive elements and structured regions (Nishikura, 2016; Bass, 2002; Nishikura, 2010). ADAR1 editing is chemically irreversible, but the dynamic synthesis and degradation of RNA molecules allow the cellular RNA pool to be continuously reshaped, making the biological effects of editing context-dependent (Rehwinkel and Mehdipour, 2025; Solomon et al., 2017). Human ADAR1 has two major isoforms: the constitutive nuclear ADAR1p110 and the interferon-inducible cytoplasmic ADAR1p150. ADAR1p110 has been reported to associated with pro-tumorigenic activity in cancers such as triple-negative breast cancer (TNBC) and hepatocellular carcinoma (HCC), suggesting it may modulate tumor pathways (Morales et al., 2022), influence the immune microenvironment, and stabilize CD24 mRNA (Sun L. et al., 2025). It has also been reported that the regulation of telomere-specific R-loops, which contribute to genome stability and cell survival of telomerase-reactivated cells (Shiromoto et al., 2021). Dysregulated R-loops can cause DNA damage, cytoplasmic nucleic acid accumulation, and type I interferon activation (Lee et al., 2025), though the direct link between ADAR1p110-regulated telomeric R-loops and IFN remains unclear. ADAR1p150 contains a unique N-terminal Z-DNA/Z-RNA binding domain (Zα) and is upregulated during viral infection or cellular stress. By editing endogenous dsRNA, it prevents activation of cytosolic sensors such as RIG-I-like receptors, acting as a negative regulator of innate immunity and distinguishing self from non-self RNA (Liddicoat et al., 2015; Mannion et al., 2014). In cancer, ADAR1p150 has been shown to play roles in distinguishing self from non-self RNA and in suppressing innate immunity, whereas much remains unknown about ADAR1p110.

## 3 ADAR1 in immune regulation and disease

Loss of ADAR1 function can lead to the accumulation of unedited endogenous dsRNA, potentially contributing to aberrant innate immune activation and the development of autoinflammatory or autoimmune diseases such as Aicardi Goutières syndrome (Rice et al., 2012). In cancer, some tumor cells depend on ADAR1 for survival (Gannon et al., 2018; Ishizuka et al., 2019). Loss of ADAR1 increases the immunogenicity of endogenous dsRNA, activating dsRNA sensors and sensitizing tumors to innate immune responses. Notably, increased expression of the ADAR1p150 isoform in tumor cells has been implicated as a central mechanism by which cancer adapts to interferon signaling and evades immune surveillance (Kung et al., 2021). While these findings underscore the roles of ADAR1 in immunity and cancer, several key questions remain regarding the context in which ADAR1 inhibition effectively sensitizes tumor cells, such as specific genetic mutations, epigenetic states, and the broader immune context, all of which have yet to be clearly defined.

## 4 RNA helicases in RNA structure remodeling and immune modulation

RNA helicases are a class of enzymes that utilize the energy derived from ATP hydrolysis to unwind RNA secondary structures and dynamically remodel RNA-protein complexes. These helicases play essential roles throughout the RNA life cycle, including transcription, splicing, translation, nuclear export, and RNA surveillance and degradation (Linder and Jankowsky, 2011). Recently, increasing attention has been directed toward their roles in the regulation of replication stress through the resolution of RNA: DNA hybrid structures known as R-loops, as well as in innate immune regulation by enhancing type I interferon (IFN-I) signaling in response to endogenous and exogenous dsRNA (Soulat et al., 2008; Chenhe et al., 2021; Szappanos et al., 2018; Choi et al., 2021; Cristini et al., 2018; Murayama et al., 2024; Mersaoui et al., 2019; Song et al., 2017; Zhang et al., 2011; Goodier et al., 2012; Skourti-Stathaki et al., 2011; Crossley et al., 2023; Miller et al., 2015). Recent studies have shown that dysregulated R-loop accumulation can lead to the activation of cytosolic sensors such as Cyclic GMP-AMP synthase (cGAS) and Toll-like receptor 3 (TLR3), activating Interferon regulatory factor 3 (IRF3) signaling and type I interferon responses (Lee et al., 2025; Crossley et al., 2023; Zannini et al., 2024) More recent work has reported that the N-terminal dsRNA-binding domain of DExH-box helicase 9 (DHX9) alone can suppress PKR activation, indicating that helicase activity may not always be required (Cottrell et al., 2024b). Together, these findings highlight the possibility that the RNA helicases employ multiple mechanisms, including direct dsRNA binding, to regulate innate immune signaling and stress responses. These degrees of functional redundancy or cooperation among distinct helicases, as well as how their subcellular localization and activity are dynamically regulated under inflammatory or tumor microenvironmental conditions, remain poorly understood.

## 5 Relationship between ADAR and RNA helicases

Recent studies have reported that ADAR1 interacts with multiple RNA helicases, regulating its RNA editing activity as well as contributing to genome stability through editing-independent mechanism. This section introduces the interactions between ADAR1 and RNA helicases.

### 5.1 Suppression of ADAR-mediated RNA editing by RNA helicases

Some RNA helicases are known to suppress ADAR-mediated A-to-I editing. For example, using an ADAR2-dependent reporter system (CREDITS and scCREDITS), DExD-Box Helicase 39B (DDX39B) was found to prevent dsRNA accumulation and act as a global repressor of A-to-I editing in a helicase- and ATPase activity-dependent manner (Wei et al., 2025). Although these findings were obtained using ADAR2 reporters, the mechanisms may also be relevant to ADAR1-mediated editing, and experimental validation using ADAR1 substrates is expected in the future. In addition, DHX9 has been reported to suppress editing of ADAR1-specific substrates in cancer cells, is overexpressed in tumors, and contributes to cancer development in a helicase activity-dependent manner (Hong et al., 2018).

### 5.2 Potential promotion of ADAR1 editing and cooperation by RNA helicases

ADAR1p110 and ADAR1p150 reside in distinct ribonucleoprotein (RNP) environments, and some of their interactions are dependent on dsRNA binding. Following interferon stimulation, core interactomes are largely maintained, but additional proximal interactions with components of antiviral stress granules are induced, providing proteomic evidence for potential editing modulators (Vukić et al., 2024). Cross-referencing these interactors with previously published RNA editing datasets suggested that several of these RNA binding proteins (RBPs) may function as modulators of A-to-I editing. However, direct functional validation remains limited (Vukić et al., 2024). In addition, DEAD-box helicase 3 X-linked (DDX3X) physically interacts with ADAR1, and dual depletion of both proteins amplifies type I interferon responses in breast cancer, such as MCF7 (Choi et al., 2021). Choi et al. showed that DDX3X can enhance ADAR1-mediated dsRNA editing in a reporter assay, although editing of endogenous RNAs was not directly tested, and further analysis is required (Choi et al., 2021).

### 5.3 Editing-independent ADAR1–helicase coordination and genome stability

Under replication stress, ADAR1 maintains genome stability through multifaced, editing-independent mechanisms. Initially, it recruits DNA Topoisomerase II Binding Protein 1 (TOPBP1) to stall replication forks, facilitating Ataxia telangiectasia and Rad3 related (ATR) activation. Upon formation of R-loops, ADAR1 relocates to these structures, relieving its inhibitory effect on TOPBP1 and further promoting ATR signaling. Finally, ADAR1 engages RNA helicases such as DHX9 and DDX21 to resolve R-loops, thereby preventing genome instability and supporting proper DNA replication (Zhang et al., 2023). Importantly, defective R-loop processing provides a direct mechanistic link to innate immune activation. Persistent RNA: DNA hybrids interfere with replication fork progression and induce replication stress, which can cause fork collapse and DNA double-strand breaks (Brickner et al., 2022). These lesions increase the formation of micronuclei, whose rupture exposes genomic DNA to the cytosol, where it is sensed by cGAS and activates Stimulator of interferon genes (STING)-dependent type I interferon responses (Crossley et al., 2023; Zannini et al., 2024). In parallel, unresolved R-loops may generate aberrant RNA species or cytosolic dsRNA that activate dsRNA sensors such as MDA5. Therefore, dysregulation of R-loop processing connects genome instability, replication stress, and cytosolic nucleic acid accumulation to innate immune activation, suggesting that the ADAR1–helicase interactions may serve as a critical checkpoint for cellular homeostasis and immune surveillance.

### 5.4 Conceptual models of ADAR1-helicase interactions

Taken together, these findings suggest the following models for the mechanisms by which ADAR1 and helicases interact.

#### 5.4.1 Model 1: RNA helicase-mediated control of ADAR substrates model

In this model, RNA helicases reduce ADAR-mediated editing by unwinding dsRNA substrates, thereby limiting duplex stability and substrate availability for A-to-I conversion. Examples include DDX39B and DHX9, which have been reported to suppress editing under specific conditions.

#### 5.4.2 Model 2: substrate remodeling and modulation model

Helicases may modulate or fine-tune ADAR1 function by remodeling RNA secondary structures, organizing RNP complexes, or shaping RNA condensates. Rather than simply competing for substrates, helicases may optimize substrate accessibility or influence spatial organization of editing complexes.

#### 5.4.3 Model 3: editing independent genome stability model

In this framework, ADAR1 cooperates with helicases independently of catalytic editing to regulate R-loop dynamics and replication stress responses. By recruiting helicases such as DHX9 and DDX21, ADAR1 is thought to contribute to the maintenance of genome stability, which may in turn indirectly influence cytosolic nucleic acid accumulation and innate immune activation.

Importantly, these models are not mutually exclusive and may coexist within the same cellular context, depending on RNA substrate features, interferon signaling status, and replication stress conditions.

## 6 Therapeutic outlook: targeting ADAR1-helicase network in cancer and innate immunity

As research on the Type I IFN signaling pathway triggered by nucleic acid recognition in tumor cells advances, its potential for cancer immunotherapy is becoming increasingly clear. By targeting the immune evasion mechanisms employed by tumors, it is possible to enhance immune responses and improve the effectiveness of existing treatments. Ultimately, overcoming the obstacles that tumors impose on innate immune pathways could lead to more effective and targeted cancer therapies.

### 6.1 ADAR1 inhibitors

Several conceptual classes of ADAR1-targeting strategies have been proposed, but these compounds may primarily act through indirect or off-target mechanisms, which could pose certain limitations fro their therapeutic application (Table 1) (O’Connell et al., 1997; Crews et al., 2023; Mendoza et al., 2023; Hang et al., 2010; Wang et al., 2025; Zhang et al., 2025; Smoak et al., 2025; Choudhry, 2021).

1. Catalytic Deaminase Inhibitors: Compounds designed to suppress A-to-I editing by mimicking adenosine substrates include nucleoside analogs such as 8-azanebularine. Additionally, Fludarabine-Cl (ZYS-1) targets the catalytic active site, but these compounds primarily act through indirect or off-target mechanisms, which may limit their therapeutic applicability (O’Connell et al., 1997; Mendoza et al., 2023; Wang et al., 2025; Zhang et al., 2025; Smoak et al., 2025).
2. RNA-Binding Domain Modulators: Compounds targeting domains such as the ADAR1p150-specific Zα domain (Alendronate, Etidronate, and Zoledronate) aim to selectively modulate interferon-inducible ADAR1 functions, but their efficacy and selectivity have not yet been experimentally evaluated (Choudhry, 2021).
3. Isoform-Specific Regulators: Agents altering the balance between ADAR1p110 and p150 isoforms, such as Rebecsinib, may enhance innate immune activation, but direct enzymatic inhibition is not yet been established (Crews et al., 2023).

**TABLE 1:** ADAR1 and RNA helicase inhibitors: recent developments, mechanisms of action, and translational challenges.

Target	Compound name	Mechanism	Translational challenges	References	Year
ADAR1p150 Zα domain	Alendronate	Targets the ADAR1p150-specific Zα domain and modulates interferon-inducible activity.	Compounds evaluated by molecular docking and molecular dynamics simulation. Not yet tested in vitro or in vivo.	Choudhry (2021)	2021
	Etidronate				
	Zoledronate				
ADAR1p150	Rebecsinib	Modulates ADAR1p150 expression through splicing inhibition.	Indirectly affects ADAR1 expression through splicing inhibition.	Crews et al. (2023)	2023
ADAR1	8-Azanebularine	It is a nucleoside analog that needs to be incorporated into ADAR1’s substrate dsRNA for use.	Inhibits ADAR1 when incorporated into nucleic acids	Mendoza et al. (2023)	2023
ADAR1 catalytic site	ZYS-1	Acts on the catalytic active site of ADAR1 to inhibit its editing activity.	The compound may have limited selectivity toward ADAR1. Effects on other targets have also been suggested.	Wang et al. (2025), Zhang et al. (2025), Smoak et al. (2025)	2025
DHX9	ATX968	Inhibition of DHX9 leads to accumulation of R-loops, which causes replication stress, induces DNA damage, and results in apoptosis selectively in MSI-H cancer cells.	Validated in vitro; in vivo studies have not yet been performed.	Castro et al. (2025)	2025
DDX3X	C1	Inhibit ATP dependent RNA helicase activity.	It's limited to in vitro enzymatic activity asssesment, and its antitumor effects have not been evaluated.	Nakao et al. (2020)	2020
DDX3X RNA binding	FHP01	Suppress Wnt/b-catenin signaling.	Validated anti-tumor effect in vitro and in vivo.	Gherardini et al. (2021)	2021

### 6.2 Helicase inhibitors

#### 6.2.1 DHX9 targeted inhibitors

As a DHX9 inhibitor, ATX968 has been identified (Castro et al., 2025). ATX968 is a small-molecule compound that selectively inhibits DHX9 and has been reported to exhibit promising antitumor effects in MSI-H/dMMR colorectal cancer models (Castro et al., 2025).

#### 6.2.2 DDX3X targeted inhibitors

C1 was identified through a screening based on ATP-dependent activity and inhibits DDX3X (Nakao et al., 2020). It regulates the unwinding of dsRNA by DDX3X, with its primary action targeting the ATP-dependent RNA helicase activity (Nakao et al., 2020). FHP01 has been identified as an inhibitor targeting the RNA-binding site of DDX3X (Gherardini et al., 2021). It suppresses Wnt/β-catenin signaling and has demonstrated anti-tumor effects in both *in vitro* and *in vivo* studies, with low toxicity (Gherardini et al., 2021).

### 6.3 Rational combination strategies

Because ADAR1 and RNA helicases play central roles in suppressing immunogenic RNA structures and aberrant nucleic acids, strategies that target both simultaneously are expected to more effectively amplify viral mimicry responses and innate immune activation than single-agent treatments. For example, pharmacological inhibition of ADAR1 using ADAR1i-124 can synergize with DNA demethylating agents such as 5-Aza-2'-deoxycytidine to increase endogenous retrovirus expression and type I interferon signaling in melanoma models (Minakuchi et al., 2026). RNA helicases, such as DHX9 and DDX3X, may modulate the availability and immunogenicity of dsRNA and aberrant RNA through RNA remodeling and interact with ADAR1 to regulate cytosolic RNA sensing (Choi et al., 2021; Cristini et al., 2018; Murayama et al., 2024; Castro et al., 2025). These findings suggest that combination strategies targeting both ADAR1 and helicase functions could be beneficial. While the effect of spindle assembly checkpoint (SAC) inhibition do not directly demonstrate cooperative regulation between ADAR1 and RNA helicases (Sasaki et al., 2025), these experiments showed that SAC inhibition leads to increased cytoplasmic dsRNA, elevated ADAR1 levels, and upregulation of dsRNA sensors. This raises the possibility that combining helicase inhibition with modulation of ADAR1 or dsRNA sensing pathways could enhance antitumor immune responses, suggesting a potential therapeutic strategy worth investigating. Future efforts should focus on defining predictive biomarkers and context-specific dependencies, as well as optimizing combination therapies, as it would be valuable to investigate RNA structural dysregulation for enhanced innate immune activation.

### 6.4 Translational challenges

However, translating ADAR1-and helicase-targeted therapies into the clinic presents several challenges. A major limitation of ADAR1 inhibitors is selectivity: many early compounds, such as N-ethylmaleimide, act nonspecifically on multiple cysteine-containing proteins (O’Connell et al., 1997), and EHNA primarily targets adenosine deaminase rather than RNA-editing enzymes (Hang et al., 2010). Nucleoside analog inhibitors depend on efficient incorporation into structured RNA substrates, which may limit their efficacy and produce widespread effects on RNA metabolism (Mendoza et al., 2023). For helicase-targeted approaches, challenges include incomplete understanding of target engagement, functional redundancy among RNA helicases, and potential toxicity associated with replication stress. Furthermore, the immune consequences of helicase or ADAR1 inhibition can vary depending on cellular context, interferon status, and baseline levels of endogenous aberrant RNA, complicating patient selection and stratification. Addressing these issues will require improved inhibitor design, predictive biomarkers, and careful evaluation of safety and context-specific effects to enable effective clinical translation.

## 7 Unresolved challenges and future directions

### 7.1 Elucidating ADAR1 functions beyond RNA editing and their impact on immune regulation

ADAR1 is well known for its RNA editing activity, but editing-independent functions and interactions with RNA helicases may also influence innate immune signaling. For instance, dsRNA binding, regulation of RNA stability may modulate intracellular immune responses independently of RNA editing. The relative contribution of editing-dependent, editing-independent, and helicase-coordinated functions likely depends on context and tumor type but remains poorly understood.

#### 7.1.1 Proposed approaches

At the cultured cell level, rescue experiments using editing-deficient or dsRNA binding-deficient ADAR1 mutants can be combined with knockdown or overexpression of related helicases to assess the contributions of editing-dependent, editing-independent, and helicase-interaction mechanisms using RNA editing profiling and dsRNA immunoprecipitation. *In vivo*, conditional genetic approaches can be used to manipulate ADAR1 and helicases in specific cell types, allowing similar analyses to clarify the effects of each mechanism within tissues or tumors.

### 7.2 Mechanistic determinants and limitations of viral mimicry activation

Activation of endogenous retroelements and ERVs can induce viral mimicry and promote anti-tumor immunity. However, excessive or sustained immune activation may potentially cause damage to cells or tissues.

#### 7.2.1 Proposed approaches

To investigate candidate repetitive RNAs associated with the ADAR1-helicase complex, RNA can be purified via co-immunoprecipitation using tagged ADAR1 and helicase, and the effects of these candidate RNAs on Interferon Stimulated genes (ISGs) expression and dsRNA sensor activation can subsequently be evaluated in cultured cells via knockdown or overexpression.

### 7.3 Identifying responsive tumor types and patient populations for ADAR1–helicase–targeted therapies

Not all tumors respond equally to ADAR1 inhibition or viral mimicry induction. Genetic backgrounds, such as p53 loss, mismatch repair deficiency (dMMR/MSI-H), or interferon pathway alterations, may determine sensitivity. Additionally, the expression and functional status of RNA helicases may influence the efficacy of ADAR1-targeted therapies.

#### 7.3.1 Proposed approaches

Integrate large-scale transcriptomic analyses (including interferon signatures, ADAR1 and helicase expression, and genomic instability markers) to identify predictive biomarkers. Functionally validate these findings using patient-derived organoids or conditional genetic models with cell type-specific manipulation of ADAR1 and helicases to strengthen stratification frameworks.

### 7.4 Differential roles of ADAR1 and helicases in tumor and immune cells

The tumor microenvironment is highly complex and ADAR1–helicase interaction may affect immune signaling and immune cell behavior. RNA modifications and helicase-mediated dsRNA remodeling can influence T cells, NK cells, macrophages, and other immune components; however, their precise impact on dendritic cell antigen presentation, macrophage polarization, or NK/T cell exhaustion, as well as their therapeutic leverage, remains unresolved.

#### 7.4.1 Proposed approaches

Apply single-cell and spatial transcriptomic profiling to investigate how ADAR-helicase interactions affect immune cell states in the tumor microenvironment. Use conditional genetic models to manipulate ADAR1 and helicases in a cell type -specific manner, clarifying their distinct roles in tumor and immune compartments.

## 8 Conclusion

In summary, ADAR1 and RNA helicases play essential roles in maintaining cellular homeostasis and innate immune responses through the regulation of RNA structure. The molecular crosstalk between these factors, including their functional interactions, represents a promising therapeutic target. The development of inhibitors with high specificity and safety may pave the way for novel epitranscriptome-based therapeutic strategies.
